# Enhancing affordance perception in pre-service physical education teachers: effects of content knowledge, motor experience and visual experience programs

**DOI:** 10.3389/fspor.2025.1583448

**Published:** 2025-07-02

**Authors:** Mariëtte van Maarseveen, Jonas Leenhouts, Annemarie de Witte, Eline Flux, Hemke van Doorn, John van der Kamp

**Affiliations:** ^1^ Research Centre for Human Movement, School and Sport & PETE Faculty, Windesheim University of Applied Sciences, Zwolle, Netherlands; ^2^ Faculty of Health, Nutrition & Sports - Research Group Healthy Lifestyle in a Supporting Environment, The Hague University of Applied Sciences, The Hague, Netherlands; ^3^ Faculty of Health, Sports, and Physical Activity; Research Group Physical Activity in and Around School, Amsterdam University of Applied Sciences, Amsterdam, Netherlands; ^4^ Faculty of Behavioral and Movement Sciences, Vrije Universiteit Amsterdam, Amsterdam, Netherlands

**Keywords:** physical education teacher education, perceptual skills, ecological psychology, teaching affordances, educational programs

## Abstract

**Introduction:**

This study examined the effects of educational programs focused on content knowledge, motor experience, and visual experience on the perceptual skills of pre-service physical education (PE) teachers. Based on an ecological approach, perceptual skill in PE teachers refers to their ability to perceive teaching affordances, i.e., the possibilities for teaching actions within the environment.

**Methods:**

A total of 60 PE pre-service teachers (age *M* = 21.0, *SD* = 2.3, 32% female) participated in educational cricket programs designed to enhance teaching skills. Participants were divided into three experimental groups (content knowledge, motor experience, visual experience) and one control group. Pre- and post-tests involved watching cricket scenarios to assess affordance perception, with gaze behavior tracked through eye-tracking technology.

**Results:**

In the post-test, participants demonstrated faster intervention times, reduced uncertainty, and a broader, more differentiated perception of affordances. This highlights improved adaptability in teaching environments. Although group differences were modest, the visual experience groups showed a larger increase in affordances related to facilitating learning than other groups. Across all groups, participants implemented more selective, impactful interventions, and relied less on verbal guidance.

**Discussion:**

These findings suggest that structured education fosters more confident, adaptive teaching styles, with small but meaningful effects depending on the type of educational content. However, the study's short duration and reliance on video-based assessments may have limited its ecological validity. This highlights the need for teacher education rooted in authentic, interactive settings. Overall, this study demonstrates the value of incorporating content knowledge, motor experience and visual experience into PE teacher education programs to enhance pre-service teachers’ perceptual skills and improve the quality of physical education teaching.

## 1 Introduction

Teaching physical education (PE) is a complex and dynamic task. In dynamic, fast-paced environments where students are constantly moving and differ widely in skills, motivation, and interests, PE teachers must manage multiple objectives simultaneously. As unexpected events frequently arise (1) PE teachers cannot rely solely on predetermined lesson plans or fixed interventions to achieve their educational goals. Instead, they must respond immediately and adaptively to the dynamic situations they encounter in order to create engaging and safe learning environments (2, 3). Quality PE thus strongly relies on a PE teacher's ability to perceive the possibilities for teaching actions that are offered by the situation.

Drawing on an ecological approach to perception (4–6), the perceptual skills of PE teachers can be understood as the ability to directly perceive possibilities for action in the teaching environment—referred to as teaching affordances (7). This approach emphasizes immediate, unreflective perception that emerges through interaction with the environment and is closely tied to an individual's goals and action capabilities. In contrast, frameworks such as the skill analysis approach (8–12) emphasize the identification of movement errors or biomechanical patterns, relying on a more analytical mode of observation. Similarly, professional vision frameworks (1, 13–17), focus on perception as a form of cognitive processing shaped by learned interpretive practices. Unlike these frameworks, the ecological approach does not separate perception from action or cognition but sees it as inherently functional and embodied—rooted in the teacher's capacity to detect actionable opportunities in real time.

For PE teachers, affordances primarily involve teaching affordances—possibilities to intervene in the lesson to direct students' behaviors toward achieving their educational goals. For example, a PE teacher might perceive the need to modify an activity to better match the students' skill level, ensuring continued engagement and learning. PE teachers are not passive observers but active participants who perceive and create relevant teaching affordances to achieve their goals (18). However, PE teachers do not perceive all possibilities indiscriminately but are drawn or attracted to those that are most relevant to their current goals. This creates a dynamic, situation-dependent field of affordances (5, 19, 20) shaped by the actions of the students, the environment, and the teacher's skills and priorities (7).

Perceptual skill, in this context, refers to a “PE teacher's tendency to perceive a more differentiated field of affordances: a broader range of affordances, extending further into the future, with greater variation in their relevance” (7). For example, a less-skilled teacher may focus solely on lesson organization by verbally guiding students through the rules of the game. In contrast, a more-skilled teacher also attends to the learning process and experiences of students, employing multiple methods to adapt the game to the students' skill levels while creating a more engaging environment. This ability to perceive a more differentiated field enables highly perceptually skilled teachers to act both flexibly and sensibly. McDonic (7) found that as pre- and in-service PE teachers gain more experience, they perceive more affordances, of a broader range and that are less predictable.

The field of affordances is dynamic, that is, affordances continuously emerge and dissolve. This field is not only influenced by the varying actions and interactions of the students and facilities, but also by the evolving concerns and skills of the teacher. Effective educational programs for PE pre-service teachers are crucial in developing the perceptual skills necessary to navigate this dynamic environment. PE teacher education (PETE) programs often incorporate strategies to deepen content knowledge and enhance motor skill or motor experience, and although PE pre-service teachers gain visual experience through their motor skill classes and internships, the curriculum less frequently contains courses that purposefully aim at gaining visual experience. To date, no studies have explored the relative contributions of content knowledge, motor experience, and visual experience to the development of perceptual skills in PE pre-service teachers, nor have these educational programs been directly compared. This explorative study aims to address this gap by examining the effects of these distinct educational programs on PE pre-service teachers' ability to perceive an increasingly differentiated field of teaching affordances. The study offers a novel contribution to understanding how different forms of experience shape perceptual skill development, aiming to provide insights into optimizing PETE to foster perceptually skilled teachers.

PE teachers' ability to perceive teaching affordances is shaped by their content knowledge (7). Content knowledge, as introduced by Shulman (21), includes the specific subject matter knowledge, understanding and skills that are to be learned by school children. Ball (22) introduced a distinction between common content knowledge (CCK) and specialized content knowledge (SCK), which Ward (23) further delineated for PE. CCK refers to the knowledge required to perform the subject, and SCK focuses on knowledge needed to teach the subject. In PE, CCK refers to knowledge about how to perform the movement or activity, including understanding rules, safety, etiquette, techniques, and tactics. SCK includes knowledge about how to represent tasks to students, sequences of instructional tasks for teaching the content, and the potential errors students might make. These forms of knowledge are not isolated; they interact and complement each other to form a comprehensive understanding that informs teaching practices.

Considerable attention has been placed on the role of content knowledge in teacher education (11, 22, 24, 25). Research highlights the importance of content knowledge in building pre-service teachers' confidence and ability to use advanced student-centered teaching strategies (26). Unlike CCK, which can be acquired through participation, SCK is typically not acquired from primary or secondary education (27), nor simply by performing an activity (28). Even repeated teaching practice over a career does not result in higher levels of SCK (28). Instead, it needs to be explicitly taught in teacher education programs (22, 23, 27–29). Studies in teacher education settings have shown that CCK and SCK scores of PE pre-service teachers can be improved through PETE content courses (24, 27, 28). For example, Cho (24) demonstrated that a seven-week volleyball content course significantly improved pre-service teachers' CCK and SCK scores, as well as their motor performance. The course included classroom instruction on instructional task sequences and error correction, as well as practical teaching sessions where they received feedback.

Studies have demonstrated that explicitly teaching SCK to teachers has causal effects on both the teachers' use of SCK in their teaching and the acquisition of motor skills by students (30–33). Nonetheless, it has not been examined if it also enhances teachers' perceptual skills.

Demonstrating and performing basic motor skills is a critical aspect of teaching PE. PETE programs therefore typically include courses designed to enhance motor experience, providing pre-service teachers with hands-on practice in various sports. In some countries, motor skill proficiency is even included in initial PETE standards (e.g., 34). Tsuda (27) have shown that PETE majors did not acquire sufficient motor skill levels prior to entering the PETE program, but their performance scores improved after taking content classes. However, the importance of personal performance as part of teaching expertise has been a subject of controversy (e.g., 25). Furthermore, the number of classes focusing on enhancing motor skills is decreasing in PETE at German universities (35). This trend is also observable in the Netherlands, where significant variations in motor skills education exist among PETE programs at different universities. This raises the question of how important performance skills are for teaching PE, in general and for perceptual skill in particular. Yet, neither the general question nor the importance of motor experience or motor skills for the perceptual skills of PE teachers has been extensively investigated.

Looking beyond the teaching domain, researchers have examined the influence of motor experience on perception. Based on common-coding theory (e.g., 36) and embodied cognition (e.g., 37), a bidirectional link between perception and action is assumed. The basic premise is that the more experienced an observer is in producing an action, the more accurately they will perceive the same action performed by another person. Support for this comes from both behavioral observations (e.g., 38, 39–41) and brain imaging studies (e.g., 42, 43). For example, Aglioti (44) found that professional basketball players perceived the outcome of basketball free throws (i.e., hit or miss) earlier and more accurately compared to coaches and sports journalists, who were presumed to have similar levels of perceptual experience but were less motorically skilled. Next to predicting movement outcomes, also in sports officiating, several studies have shown that motor experience positively influences the quality of perception and decision-making accuracy (45, 46). Research indicates that perceptual judgements can even be enhanced by motor training alone (39, 47, 48). For instance, in a study by Casile and Giese (39), participants who learned novel and unusual arm movements while blindfolded, showed improved visual discrimination of the previously learned movements in a visual discrimination task.

In PETE settings, studies have shown that pre-service teachers' motor skills can be improved through structured content courses (24, 27). To the authors' knowledge, the only study examining the effects of motor experience on PE teachers' perception is the study by Reuker (35). Using the professional vision framework, she compared observations and interpretations of students' activities in a basketball PE lesson among groups with different sport-specific and pedagogical expertise. The two groups with high sport-specific expertise (one group with only high sport-specific expertise and the other with both high sport-specific and pedagogical expertise) did not differ from the teachers (who were considered to have only high pedagogical expertise) in terms of the attention paid to motor learning. The only motor experience-related difference was that the expert group (with both high sport-specific and pedagogical expertise) focused on methodological aspects more often than the teacher group (with only high pedagogical expertise). However, the task required participants to watch video clips of PE lessons and report significant events after each clip. This means they were asked to report what they noticed as important for teaching practices, not what they would do in that specific situation. Therefore, it remains unclear what the role of motor experience is in perceiving teaching affordances by a PE teacher.

Perception is influenced not only by the specific situation but also by one's prior visual experiences (45, 47). Research in the medical field demonstrates that experience and deliberate practice significantly enhances medical image interpretation skills (49). Similarly, studies in sports officiating reveal that performance improves with increased visual experience, such as watching games in stadiums or on television (37). In teacher education, skill analysis approaches have shown that perceptual training effectively enhances error detection during video-based assessments. These training interventions typically involve pre-service teachers watching videos containing correct and incorrect movement patterns selected by researchers or experts (50, 51), or practicing error detection on videos and receiving feedback on their accuracy (52–54). Surveys have shown that most PE pre-service teachers report having no dedicated training in skill analysis. Consequently, Ward (11) concluded that enhancing the deliberate practice of skill analysis in PETE programs is the key to addressing inaccurate movement error perception. Using the professional vision framework, Sherin and Van Es (13, 14, 16) found that mathematics teachers' noticing can be developed through video clubs, where groups watch and discuss video clips of teaching situations, thus increasing their visual experience. Additionally, Gold (55) showed that video-based learning improved the professional vision of classroom management among pre-service teachers (not PE). However, identifying actions or errors in movement patterns is fundamentally different from actively teaching in dynamic environments. As a result, the affordances perceived while watching videos are likely distinct from those perceived during real-time teaching (56, 57). The influence of visual experience on affordance perception of PE teachers has not been experimentally investigated yet.

This study investigates the effects of targeted education in content knowledge, motor experience, and visual experience on PE pre-service teachers' perceptual skill, when measured as differentiation in the field of teaching affordances. The study hypothesizes that, following education, pre-service teachers will perceive a greater quantity, diversity and unpredictability of teaching affordances, indicative of a more differentiated field of affordances. However, the literature provides no basis for preconceived expectations about differences in effects between the targeted educational programs. PE pre-service teachers participated in a six-week cricket course. Before and after the course, they watched videos of cricket activities and verbally reported the teaching affordances they perceived. The quantity, diversity and unpredictability of the perceived teaching affordances were compared across time and between groups to assess the development of perceptual differentiation. Additionally, gaze behavior was measured to explore whether differences in the perception of teaching affordances were underpinned by differences in gaze behavior.

## 2 Materials and methods

### 2.1 Study design

A pre-post, quasi-experimental design was conducted in adherence to the guidelines outlined in the CONSORT statement (58) (see Supplementary Table S1). Ethical approval for the study was obtained from the Scientific and Ethical Review Committee at the Vrije Universiteit Amsterdam.

### 2.2 Participants

In total 60 PE pre-service teachers were recruited from two PETE faculties in the Netherlands. These participants were in their second, third or fourth (final) year of teacher training (bachelor degree), and all had normal or corrected-to-normal vision. Individuals with prior cricket or coaching experience were excluded. Of the 60 participants, 45 enrolled in an elective cricket teaching course, for which they received European Credits upon successful completion. Participants were assigned to one of three experimental groups—Content Knowledge, Motor Experience or Visual Experience—based on their PETE faculty. A control group, consisting of 15 PETE students from the same faculties completed only pre- and post-tests and did not receive European Credits. As an incentive, participants in the control group received a 30 euro voucher for a reputable online store.

Table 1 presents the demographic details of each group. Group allocation was determined by practical considerations related to institutional scheduling and curriculum organization. Although the assignment was not fully randomized, efforts were made to ensure baseline comparability across groups. Nonparametric analysis of pre-test scores indicated no significant differences between faculties, all *p*s > .05, supporting the validity of comparisons across conditions. Written informed consent was obtained from all participants prior to their involvement in the research.

**Table 1:** Demographic characteristics of participant groups.

Group	*n*	Female *n* (%)	Male *n* (%)	Age Years (SD)
Content knowledge	15	4 (26.7)	11 (73.3)	21.3 (2.2)
Motor experience	14	4 (28.6)	10 (71.4)	22.0 (3.2)
Visual experience	16	5 (31.2)	11 (68.8)	20.6 (2.0)
Control	15	6 (40.0)	9 (60.0)	20.3 (1.6)
All	60	19 (31.7)	41 (68.3)	21.0 (2.3)

### 2.3 Materials

To assess affordance perception, a series of video clips was created depicting children playing cricket games and drills. Cricket was chosen because it is seldom played in the Netherlands and rarely featured on television. It is almost never taught in PE lessons, ensuring that all pre-service teachers had similar entry levels. The recordings were conducted at two secondary schools and during a regional cricket training session. At one secondary school, filming took place in a second grade PE class, led by their PE teacher renowned for his expertise and passion in teaching cricket. These students had received three to four cricket lessons and were moderately familiar with the sport. In contrast, the first-grade students at the second school had no prior exposure to cricket and participated in an introductory lesson conducted by an experienced coach from the Royal Dutch Cricket Association. Additional recordings were made at a regional cricket club in The Hague, capturing a training session involving children aged 6–12. Written informed consent was obtained from all children and their parents before filming.

All sessions were recorded using three or four digital video cameras placed at eye-height viewpoints to provide a teacher's first-person perspective. We selected video fragments with little or no teacher intervention to maintain authenticity and minimize bias. This resulted in 36 video clips, some capturing the same students and activities from different angles. The video clips included indoor batting, bowling and fielding drills, as well as various adapted cricket games. Subsequently, the research team reviewed the footage and selected 10 representative clips that depicted activities suited for PE lessons, typical student behavior, and a range of variations in activities, locations, and the proficiency levels of the children. The duration of these clips ranged from 1 min 16 s to 7 min 10 s.

A monitor with a 24-in screen and a keyboard were used to display the experimental instructions, stimuli (i.e., video clips) and post-test questionnaire and record the typewritten responses. Tobii Pro Lab software (version 1.118) was used for stimulus presentation, response registration, and eye tracking. The Tobii Pro Nano screen-based eye tracker recorded participants' gaze behaviors during video clip observation. Calibration of the eye tracker was conducted using the standard calibration and validation function on 5 targets. The eye tracker records with a sampling frequency of 60 Hz, a precision of 0.20° and an accuracy of 0.3°. It operates within a distance of 45–85 cm and allows freedom of head movement within 35 × 30 cm (width, height) from a distance of 65 cm. A Yeti Pro microphone was used to record the verbal responses of the participants.

The post-test questionnaire, designed in Qualtrics (Provo, UT), aimed to evaluate participants' cricket-specific perceived motor competence and content knowledge. Participants rated their own cricket performance on a scale of 0–10, even if they had never played it. Content knowledge was evaluated through a 24-item multiple-choice test developed in collaboration with PETE games teachers and the Royal Dutch Cricket Association, and based on PE subject matter knowledge domains described by Ward (23). Fourteen test items focused on common content knowledge, including knowledge of rules, etiquette, techniques and tactics for playing cricket. The remaining ten items covered specialized content knowledge, including teaching methodology, instructional tasks, common mistakes, and educational support.

### 2.4 Procedure and intervention

Before and after the educational program, the PE pre-service teachers underwent an identical perception test. Participants sat at a desk in a quiet room, facing a monitor. After calibrating the eye tracker, they read the instructions on the monitor and could ask the researcher for clarification. Participants were instructed to imagine themselves teaching the children depicted in the video clips and to provide verbal guidance as they would in a real PE setting. This verbal guidance reflects participants' perception of affordances for guiding. When participants wished to intervene during the activity, that is to interrupt the activity of the children, they paused the video by pressing the space bar and then reported the action(s) or teaching intervention(s) they would undertake. These perceptions concern the affordances for intervention. The recorded verbalizations, both during video playback and after pausing it, were used to identify perceived teaching affordances offline. During an initial familiarization trial, participants could ask questions, with the experimenter offering further clarifications as needed. If the participant remained silent or did not intervene for more than two minutes, the experimenter repeated the instructions. Following confirmation of task clarity, nine trials were presented. Each trial began with a screenshot of the video clips' initial frame and the activity's rules. Participants initiated video playback by pressing the space bar. Throughout the experimental task, participants' gaze behaviors and verbal responses were recorded. Finally, participants completed the post-test questionnaire. The entire test, including the questionnaire, lasted approximately 45–60 min.

The educational program comprised six lessons of two hours each. The first lesson was consistent across all intervention groups, featuring a general introduction to cricket, containing various introductory drills and adapted games. The subsequent five lessons consisted of an intervention-specific part (1 h) followed by a general part (1 h). In the intervention-specific part, lessons aimed to either enhance Content Knowledge (CK), Motor Experience (ME) or Visual Experience (VE). The Content Knowledge group lessons, developed in collaboration with an experienced PETE games teacher, focused on enhancing understanding of cricket in PE, covering rules and terminology, techniques, tactics, biomechanics, game balance, didactics, and methodologies. The Motor Experience group lessons, led by an experienced trainer of the Royal Dutch Cricket Association, focused on improving motor skills through drills in batting, bowling, fielding, running, and their application in small-sided cricket games. The Visual Experience group acquired cricket-specific visual experience by watching video footage of various cricket drills, adapted games and official matches. Assignments included tasks related to rules, game balance, technique, tactics, didactics and methodology. For instance, they watched six children performing a batting drill and had to rank their performance or they watched children playing a small-sided cricket game and had to identify good and bad strategic moments or decisions. Video footage for these lessons included recordings of PE lessons (distinct from those used in pre- and post-tests), Dutch youth national team training sessions, lessons from the Content Knowledge and Motor Experience groups, World Cup (2024) matches and YouTube videos.

In the general part of the educational program participants were assigned tasks to teach their peers adapted cricket games. The series of tasks began with easy games with simple rules (aimed at primary school children), continued with specific batting, bowling and fielding drills and concluded with small-sided cricket games (suited for secondary school). Participants were challenged to design rich learning environments, manipulate constraints in order to get a good balance between bowler, batter(s) and fielders, in order to facilitate learning and foster enjoyable learning experiences for players. Participants received feedback on their teaching practices from their peers and from the teacher of the educational program. Fidelity was ensured through standardized lesson plans and a shared intervention guide, both of which were co-developed by all teachers involved in teaching the program. All teachers followed these shared materials, and peer feedback during general sessions contributed to ongoing monitoring. All sessions were delivered as planned, with no reported deviations from the protocol. Table 2 summarizes both the general and intervention-specific components of the educational program using the TIDieR checklist (59, 60).

**Table 2:** TIDieR checklist – intervention summary.

Item no	Item	Details			
1	Brief name	General Part	Content Knowledge	Motor Experience	Visual Experience
2	Rationale/Goal	Gain baseline understanding of cricket and develop teaching skills through peer-teaching tasks.	Enhance common and specialized content knowledge on teaching cricket.	Improve motor competencies and embodied understanding of cricket skills.	Enhance visual experience by watching cricket videos.
3	Materials	Workbook with peer-teaching tasks and adapted game design assignments.	Workbook with assignments; covering cricket (teaching) theory.	Cricket equipment.	Workbook with structured video-based tasks. Video footage included recordings of PE(TE) lessons, Dutch national youth team training sessions, World Cup 2024 matches, YouTube videos.
4	Procedures	Initial 2-hour introductory session on cricket, containing various drills and adapted games. Then, 5 weekly 2-hour sessions with 1 h of intervention-specific instruction (content knowledge, motor experience or visual experience) and 1 h of general cricket teaching. In this general part, participants were assigned tasks to teach their peers adapted cricket games. The series of tasks began with easy games with simple rules (aimed at primary school children), continued with specific batting, bowling and fielding drills and concluded with small-sided cricket games (suited for secondary school). Participants were challenged to design rich learning environments, manipulate constraints in order to get a good balance between bowler, batter(s) and fielders, in order to facilitate learning and foster enjoyable learning experiences for players. Participants received feedback on their teaching practices from their peers and from the teacher of the educational program.	The Content Knowledge group lessons, developed in collaboration with an experienced PETE games teacher, focused on enhancing understanding of cricket in PE. The program covered key topics such as rules and terminology, techniques, tactics, biomechanics, game balance, didactics, and methodologies. For instance, participants completed fill-in-the-blank exercises on fundamental cricket rules, researched and described key technical points for batting, bowling, and fielding, and ranked adapted games from simple to complex.	The Motor Experience group lessons, led by an experienced trainer of the Royal Dutch Cricket Association, focused on improving motor skills through practical drills in batting, bowling, fielding, running, and their application in small-sided cricket games. For instance, participants received instruction on bowling technique and practiced bowling with gradually increasing speed, or worked on batting drills aimed at directing the ball to specific areas of the field.	The Visual Experience group acquired cricket-specific visual experience by watching video footage of various cricket drills, adapted games and official matches. Assignments included tasks related to rules, game balance, technique, tactics, didactics and methodology. For instance, they watched six children performing a batting drill and had to rank their performance or they watched children playing a small-sided cricket game and had to identify good and bad strategic moments or decisions.
5	Who provided	Experienced PETE games teacher.	Experienced PETE lecturer.	Experienced and certified coach from Royal Dutch Cricket Association.	Experienced PETE lecturer.
6	How (mode of delivery)	Live, group format.	Live, group format.	Live, group format.	Live, group format.
7	Where	Gymnasium (sports hall).	Classroom.	Gymnasium (sports hall).	Classroom.
8	When and How Much	6 sessions: One initial 2-hour session; then 5 weekly 1-hour sessions.	1 h/week for 5 weeks.	1 h/week for 5 weeks.	1 h/week for 5 weeks.
9	Tailoring	Instructional feedback was tailored to students’ progression throughout the program. However, no formal individualization protocol was used.	Instructional feedback was tailored to students’ progression throughout the program. However, no formal individualization protocol was used.	Instructional feedback was tailored to students’ progression throughout the program. However, no formal individualization protocol was used.	Instructional feedback was tailored to students’ progression throughout the program. However, no formal individualization protocol was used.
10	Modifications	No modifications occurred during the intervention.	No modifications occurred during the intervention.	No modifications occurred during the intervention.	No modifications occurred during the intervention.
11	Planned Fidelity Assessment	Fidelity was ensured through standardized lesson plans and a shared intervention guide, both of which were co-developed by all teachers involved in the program. All teachers followed these shared materials, and peer feedback during general sessions contributed to ongoing monitoring.	Fidelity was ensured through standardized lesson plans and a shared intervention guide, both of which were co-developed by all teachers involved in the program. All teachers followed these shared materials, and peer feedback during general sessions contributed to ongoing monitoring.	Fidelity was ensured through standardized lesson plans and a shared intervention guide, both of which were co-developed by all teachers involved in the program. All teachers followed these shared materials, and peer feedback during general sessions contributed to ongoing monitoring.	Fidelity was ensured through standardized lesson plans and a shared intervention guide, both of which were co-developed by all teachers involved in the program. All teachers followed these shared materials, and peer feedback during general sessions contributed to ongoing monitoring.
12	Actual Fidelity	All sessions were delivered as planned, with no reported deviations from the protocol. Attendance and instructor logs confirmed adherence.	All sessions were delivered as planned, with no reported deviations from the protocol. Attendance and instructor logs confirmed adherence.	All sessions were delivered as planned, with no reported deviations from the protocol. Attendance and instructor logs confirmed adherence.	All sessions were delivered as planned, with no reported deviations from the protocol. Attendance and instructor logs confirmed adherence.

Note: Based on the TIDieR checklist (59).

### 2.5 Data analysis

A quantitative content analysis was employed to explore the perceived teaching affordances and differences among groups and pre and post—tests. Audio recordings of participants' responses were transcribed verbatim and pseudonymized. Participants' statements during video playback (guiding affordances) and after pausing (intervention affordances) were coded. A unit of analysis was defined as one separate statement of a participant, varying in length from one word to several sentences. Statement could include multiple teaching affordances, but repeated mentions of the same type within a statement were counted once. Coding was performed using Atlas.ti software (Windows version 22.2.4.0). A coding scheme was developed using content analysis, combining inductive and deductive strategies and yielding quantitative frequencies for analysis (61–63). A first version of a coding scheme was collectively developed by closely analyzing participants' verbalizations as well as building on the coding scheme of McDonic (7). When applicable, terminology from existing scientific and educational literature was adopted to label and define codes. Through iterative rounds of coding, codes were adapted and merged until consensus was reached, resulting in a coding scheme comprising codes and descriptions, as detailed in Table 3. To establish coding reliability, four researchers independently coded ten randomly selected transcripts. Intercoder reliability was assessed using Krippendorff's c-Alpha-binary, resulting in an overall score of 0.866, indicating good reliability. All codes were categorized into Managing Lessons, Facilitating Learning or Guiding Experiences, which represents a well-established didactical model for PE in The Netherlands (64). Teaching interventions in the category Managing Lessons aim at organizing the lesson; those aim to improve the degree to which the behavior of the children is orderly, or children are even able to self-regulate the activity. Teaching interventions in the category Facilitating Learning aim at enhancing the (motor) performance of the children, and their possibilities to explore and experiment with motor solutions. Teaching interventions in the category Guiding Experiences aim at enhancing children's engagement and motivation for the activity or lesson.

**Table 3:** Coding scheme.

Category	Code	Definition
Managing lessons	Adjusting activity	Changes to the present activity (e.g., equipment, rules, distances) to make it run smoother or for safety reasons, for example: increase distance between the bowler and wicket, saying “don’t hit the cones with the bat”
	Directing attention	Directing the attention of the student(s) to the activity without specifying a particular action, for example: pay attention everyone, are you ready?
	Other activity	Choosing a completely different activity because the current one is not running well
	Coaching	Verbal cues, comments, instructions indicating what the player(s) should do immediately, specifying an action, for example: run now, catch the ball, that's out, etc. without specifying how to do it
	Correction behavior	Actions to correct the behavior of the student(s), for example: addressing them, giving a consequence
	Teacher participation	The teacher participates in the activity to make it run smoother
	Repeating	Instructing the student(s) to repeat the same action immediately
	Organizing lesson	Adjustments to the entire activity/lesson to make it run smoother or for safety considerations, for example: more distance between groups
	Clarifying rules	Clarifying the rules by repeating, explaining differently, or demonstrating to make the activity run smoother, both game rules and lesson rules
	Organizing lesson inquiry-based	Improving the flow of the activity by asking students questions about the activity/rules/goals/etc., for example: where should you run after hitting the ball?
Facilitating learning	Adjusting activity	Adjustments to the activity (rules, distances, players, etc.) to make it more successful, for example: place the bowler closer to the wicket, place the cone further away, bowl underarm
	Other activity	Choosing a completely different activity because the current one is not succeeding well
	Demonstration	A demonstration by the teacher or another student to show students how to perform the activity/the movement correctly, to make it more successful
	Teacher participation	Teacher participates in the activity to make it more successful or to help students
	Feedback negative	Statements specifically intended to address what is not going well in the technical execution or tactics of the activity, specifying an action, for example: you're holding the bat the wrong way or you didn’t collaborate well
	Feedback positive	Statements specifically intended to address what is going well in the technical execution or tactics of the activity, specifying an action, for example: you're hitting the ball nicely with the lower part of the bat or well spotted that you could run again
	Manual learning support	Helping the student(s) by physically moving the student's body into the correct position/movement
	Tip	Tips/instructions intended to improve the technical execution or the tactical execution/strategy on the next attempt, for example: stand sideways when batting or keep an eye on the fielders to see if you can run again
	Learning support inquiry-based	The teacher asks questions to prompt the student(s) to think about how the technical execution or the tactics of the activity can be improved, for example: how can you hit the ball harder or what is smart when running?
Guiding experiences	Encouragement	Expressing encouragement to motivate students, for example: come on, yes, go, go, go, keep going, oh too bad next time better
	Adjusting activity	Adjustments to the activity (rules, distances, players, etc.) to make the activity more engaging, for example: keeping score
	Other activity	Choosing a completely different activity because the current one is not engaging well
	Expressing disapproval	Negative or demotivating statements indicating the teacher's dissatisfaction and not specifically aimed at improving the player(s)’ movement, for example: damn, what a mess
	Giving compliment	Positive or motivating statements indicating the teacher's satisfaction, and not specifically aimed at improving the player(s)’ movement, without specifying an action, for example: well done, nice, great job
	Teacher participation	Teacher participates in the activity to make it more engaging
	Jokes	The teacher makes a joke or comment intended to make the student(s) laugh or smile
	Inquiry-based guiding experiences	The teacher asks questions to prompt the student(s) to think about how the activity could be made more engaging, for example: what could we do to make it even more fun?
	Expressing concerns	Actions to inquire about or influence the well-being of the student(s) or express concerns, for example: I would check in with that girl over there to see what's wrong, why she's sitting there
Other	Uncertainty	Participant expresses doubts or uncertainty about what to do

To identify differences in perceived teaching affordances among groups and tests, the time until participants paused the video for intervention (hereafter referred to as time to intervention), and the frequencies of the codes per participant, type (guiding affordances and intervention affordances) and test were exported to Excel and R for further quantitative analysis. The quantity of teaching affordances was measured as the total number of affordances, and diversity was measured as the number of unique teaching affordances and as Shannon entropy. This latter is a measure of the degree to which there is structure or predictability in the teaching affordances perceived (65). Low entropy scores reflect high predictability, and high entropy scores mean less predictability. In the gaze behavior data, fixations were identified using the Tobii Pro Lab gaze filter “Attention”. Search rate (mean number of fixations per second) and mean fixation duration were calculated for each participant and test.

### 2.6 Statistical analysis

Data were analyzed using R software. Because the data were not normally distributed, the Aligned Rank Transform (ART) method was used (66). To examine differences in perceived teaching affordances, independent 4(Group: Content Knowledge, Motor Experience, Visual Experience, Control) × 2 (Test: Pre, Post) × 2 (Type: Guiding affordances, Intervention affordances) ART ANOVAs with repeated measures on the last two factors were conducted on the total number of perceived teaching affordances, the number of unique affordances, and Shannon's Entropy scores. The time to intervention was examined with a Group X Test ART ANOVA with repeated measures on the last factor. The distribution of the teaching affordances across the didactical categories was analyzed using a 4 (Group: Content Knowledge, Motor Experience, Visual Experience, Control) × 2 (Test: Pre, Post) × 3 (Category: Managing lessons, Facilitating learning, Guiding experiences) ART ANOVAs with repeated measures on the last two factors. The gaze behavior variables search rate and fixation duration were analyzed with two separate ART ANOVAs with group as between-factor and test as within-factor. Also, the educational program related measures, perceived motor skills, CCK and SCK were analyzed with independent Group x Test ART ANOVAs with repeated measures on the last factor. *post hoc* tests used the emmeans package with Tukey's HSD adjustment for multiple comparisons. Partial eta squared (*η_p_^2^*) values and 95% confidence intervals were calculated to estimate effect sizes and indicate the reliability of the observed effects. Only statistically significant pairwise comparisons are reported here for clarity; see Supplementary Table S2 for all pairwise comparisons.

## 3 Results

See Table 4 for all statistics.

**Table 4:** Statistical overview.

DV	Term	Df	Df.res	*F*	*p*	*η_p_^2^*
Time to intervention						
Group		3	56	2.366	0.081	0.113
Test		1	56	36.056	<.001	0.392
Group x Test		3	56	1.282	0.290	0.064
Number of perceived teaching affordances						
Group		3	56	2.963	0.040	0.137
Test		1	56	58.901	<.001	0.513
Type		1	56	18.669	<.001	0.250
Group x Test		3	56	3.875	0.014	0.172
Group x Type		3	56	4.288	0.009	0.187
Test x Type		1	56	60.870	<.001	0.521
Group x Test x Type		3	56	2.395	0.078	0.114
Number of unique teaching affordances						
Group		3	56	0.943	0.426	0.048
Test		1	56	2.989	0.089	0.051
Type		1	56	0.368	0.547	0.007
Group x Test		3	56	0.524	0.667	0.027
Group x Type		3	56	1.905	0.139	0.093
Test x Type		1	56	18.995	<.001	0.253
Group x Test x Type		3	56	0.377	0.770	0.020
Shannon entropy						
Group		3	45	2.199	0.101	0.128
Test		1	45	0.554	0.461	0.012
Type		1	45	1.453	0.234	0.031
Group x Test		3	45	2.060	0.119	0.121
Group x Type		3	45	2.663	0.059	0.151
Test x Type		1	45	8.307	0.006	0.156
Group x Test x Type		3	45	0.867	0.465	0.055
Teaching affordance didactical categories						
Group		3	56	0.979	0.409	0.050
Test		1	56	1.074	0.305	0.019
Category		2	112	27.499	<.001	0.329
Group x Test		3	56	0.707	0.552	0.037
Group x Category		6	112	2.665	0.019	0.125
Test x Category		2	112	12.017	<.001	0.177
Group x Test x Category		6	112	2.369	0.034	0.113
Uncertainty						
Group		3	56	1.743	0.169	0.085
Test		1	56	34.066	0.000	0.378
Group x Test		3	56	1.190	0.322	0.060
Search rate						
Group		3	56	1.851	0.148	0.090
Test		1	56	7.658	0.008	0.120
Group x Test		3	56	1.523	0.219	0.075
Fixation duration						
Group		3	56	0.953	0.421	0.049
Test		1	56	17.512	<.001	0.238
Group x Test		3	56	0.999	0.400	0.051
Motor skills						
Group		3	56	4.185	0.010	0.183
Test		1	56	50.034	<.001	0.472
Group x Test		3	56	9.283	<.001	0.332
Common content knowledge						
Group		3	56	0.457	0.713	0.024
Test		1	56	3.653	0.061	0.061
Group x Test		3	56	0.840	0.478	0.043
Specialised content knowledge						
Group		3	56	1.774	0.163	0.087
Test		1	56	4.190	0.045	0.070
Group x Test		3	56	1.440	0.241	0.072

### 3.1 Affordance perception

#### 3.1.1 Time to intervention

**Figure 1:**
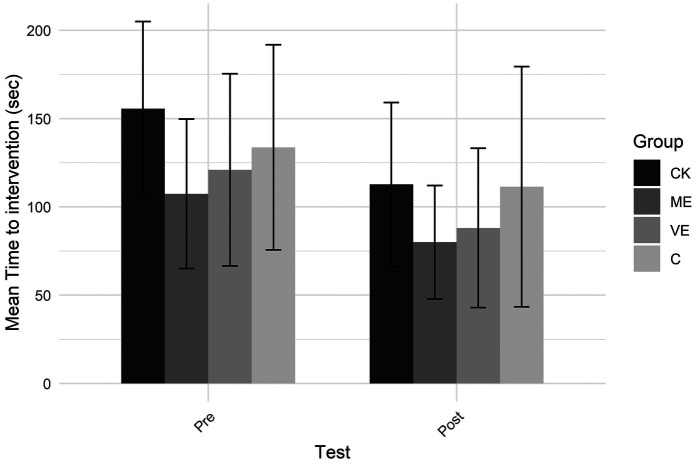
Time to intervention.

The time to intervention for each group and test is shown in Figure 1. Only a significant main effect of test was found, indicating that participants intervened earlier in the post-test than in the pre-test.

#### 3.1.2 Number of perceived teaching affordances

**Figure 2:**
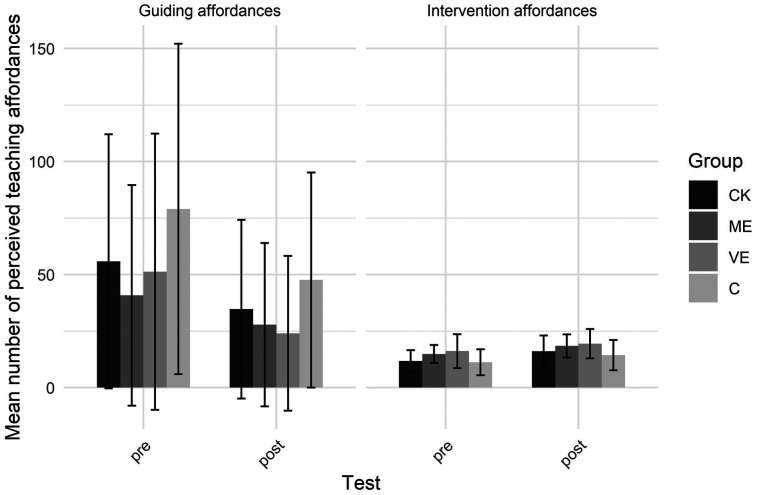
Number of perceived teaching affordances.

Figure 2 shows the number of perceived teaching affordances for each group, type and test. A significant group by test interaction indicated that changes in perceived affordances over time differed by group. Specifically, only the ME group showed a significant increase in their total number of perceived teaching affordances from pre to post-test. The CK and VE groups showed no changes, while the C group trended toward a decrease in perceived teaching affordances. The group by type interaction indicated no significant differences between groups in guiding affordances. However, the C group perceived significantly fewer intervention affordances than the ME and VE groups. Both the ME and VE groups perceived relatively more intervention affordances than guiding affordances. The test by type interaction showed that across groups, participants decreased the number of guiding affordances from pre to post test and increased the number of intervention affordances.

Furthermore, a significant main effect of test revealed that more affordances were perceived in the pretest than in the post test. A significant main effect of type showed that more guiding affordances were perceived than intervention affordances. And a significant main effect of group showed that the ME group perceived less affordances than the C group.

#### 3.1.3 Diversity in teaching affordances

**Figure 3:**
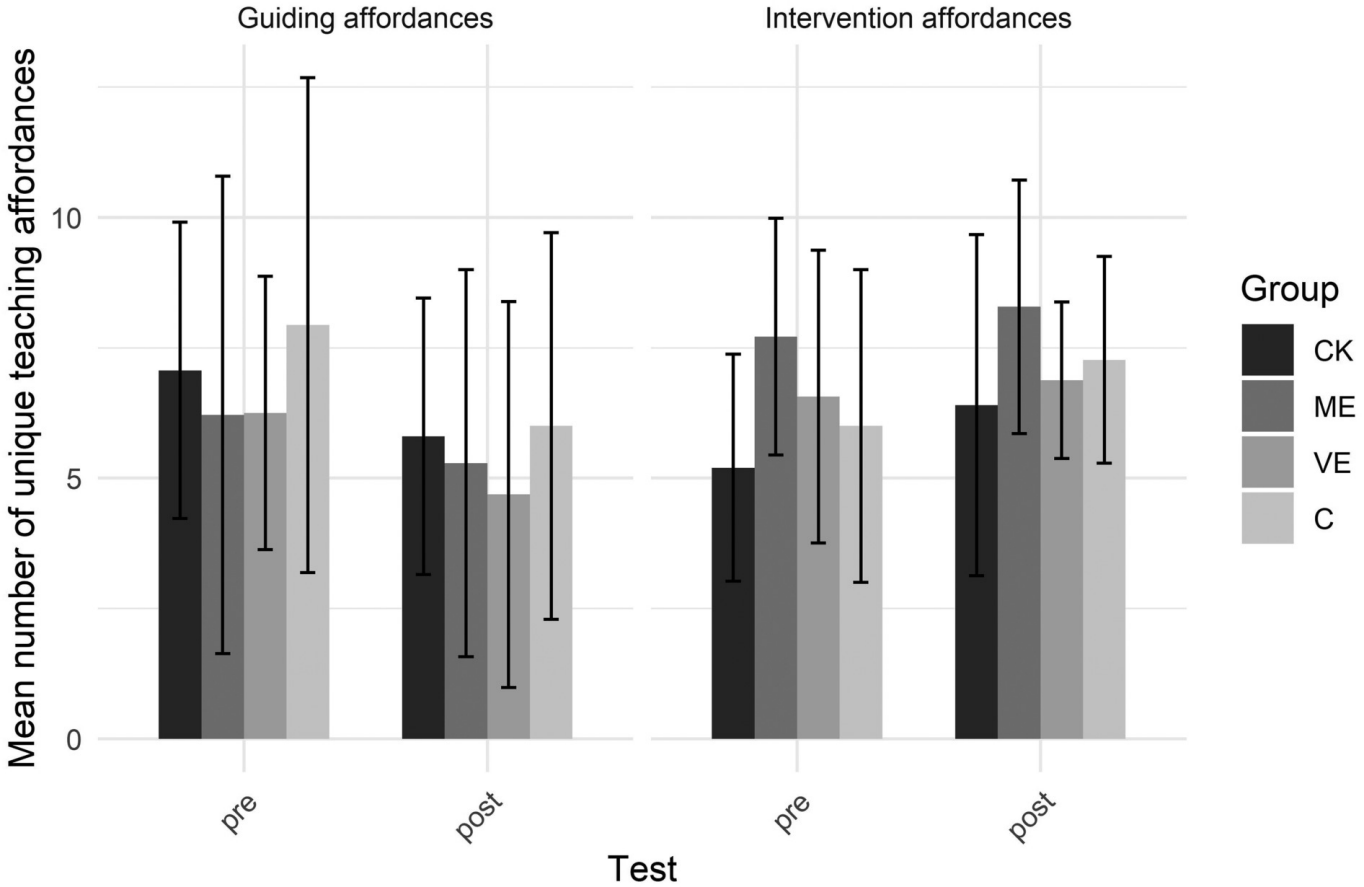
Number of unique teaching affordances.

**Figure 4:**
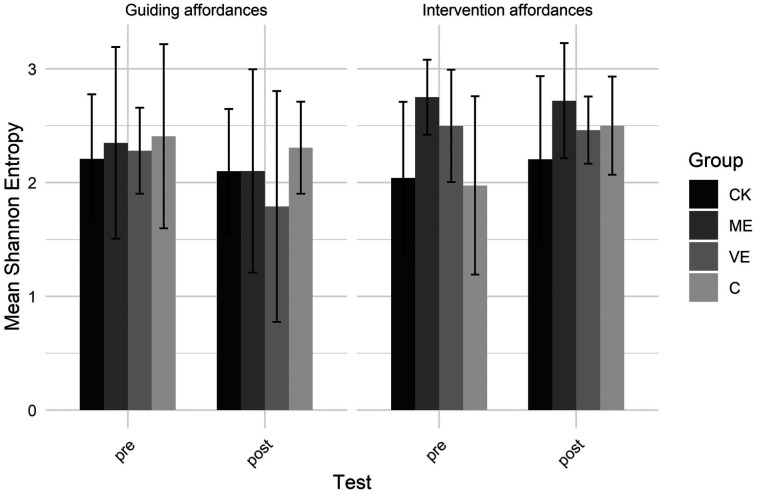
Shannon entropy.

Figures 3, 4 show the number of unique teaching affordances and entropy scores, respectively. A significant test by type interaction showed unique guiding affordances decreased and unique intervention affordances increased from pre- to post-test. For Shannon entropy, a test by type interaction showed decreased entropy for guiding affordances, indicating they became more predictable during the post-test, and increased entropy for intervention affordances, indicating less predictable intervention affordances during the post-test.

#### 3.1.4 Teaching affordance didactical categories

**Figure 5:**
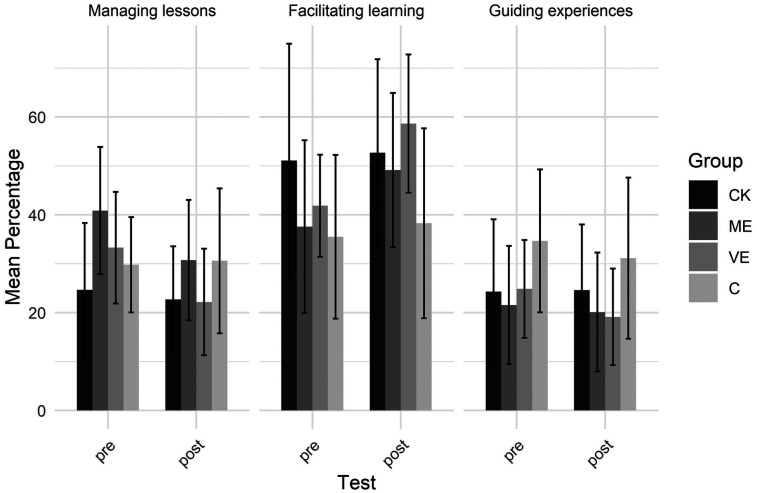
Teaching affordance didactical categories.

Figure 5 shows the distribution of the teaching affordances across the three didactical categories: managing lessons, facilitating learning, and guiding experience. A significant group by test by category interaction effect showed that the VE group increased the percentage teaching affordances categorized as facilitating learning from pre to post-test. They also marginally decreased those categorized as managing lessons. Notably, the C group tended to increase the percentage teaching affordances categorized as managing lessons from pre to post-test.

Across groups, a significant test by category interaction revealed facilitating learning affordances increased, while managing lessons affordances decreased from pre- to post-test. At the pre-test, managing lessons affordances outnumbered facilitating learning, but this difference disappeared by the post-test. A significant group by category interaction effect showed that the C group perceived more affordances for guiding experiences than for facilitating learning. Their percentage of facilitating learning affordances was also lower than that of the VE group. Overall, more affordances were categorized as facilitating learning than as managing lessons or guiding experiences (main effect of category).

#### 3.1.5 Uncertainty

**Figure 6:**
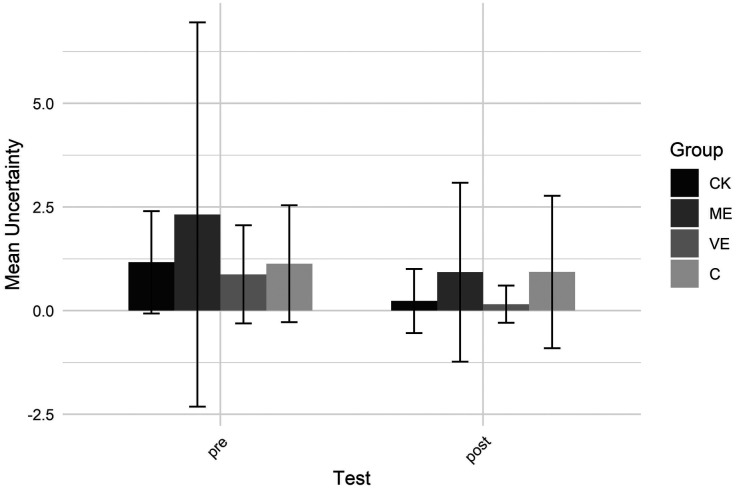
Uncertainty.

While analyzing the data, we found that participants regularly expressed doubts or uncertainty about what to do while watching the video clips, see Figure 6. An additional Group x Test ART ANOVA was conducted on the mean number of uncertainty statements to examine changes over time. A significant main effect of test showed that participants expressed less uncertainty, thus were more self-confident, in the post-test than in the pre-test.

### 3.2 Gaze behavior

**Figure 7:**
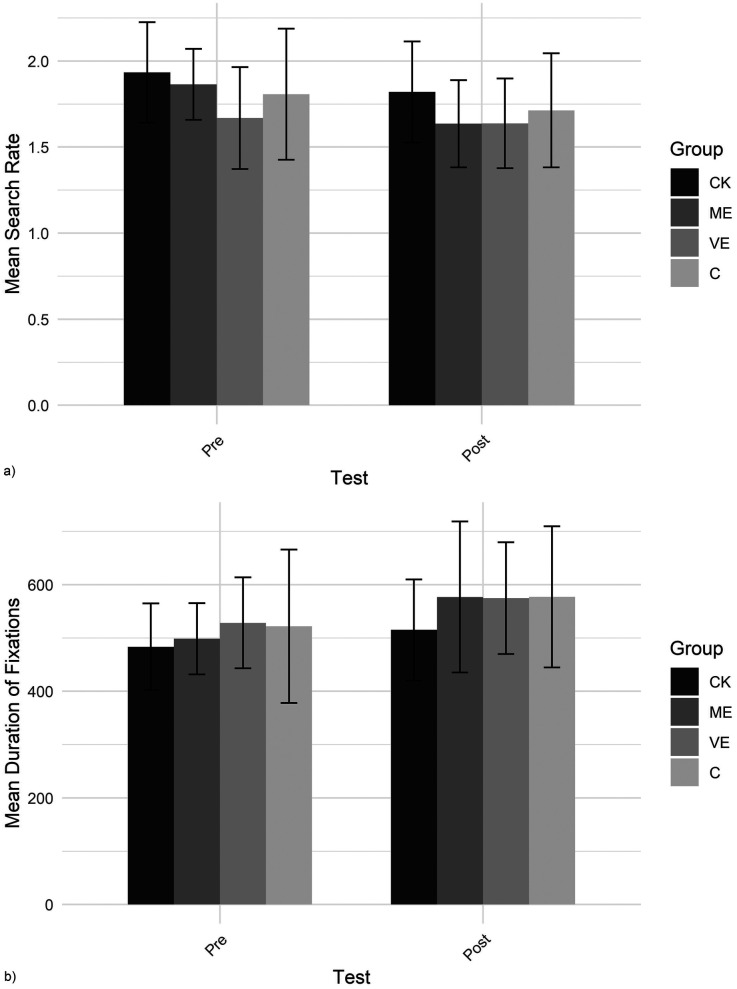
Gaze behavior: **(a)** search rate and **(b)** fixation duration.

The search rate and fixation duration are shown in Figure 7. For *search rate,* only a significant main effect of test was found, showing a lower search rate in the post-test than in the pre-test. For *fixation duration,* only a significant main effect of test was found, indicating longer fixations in the post-test than in the pre-test.

### 3.3 Educational program related measures

#### 3.3.1 Perceived motor skills

**Figure 8:**
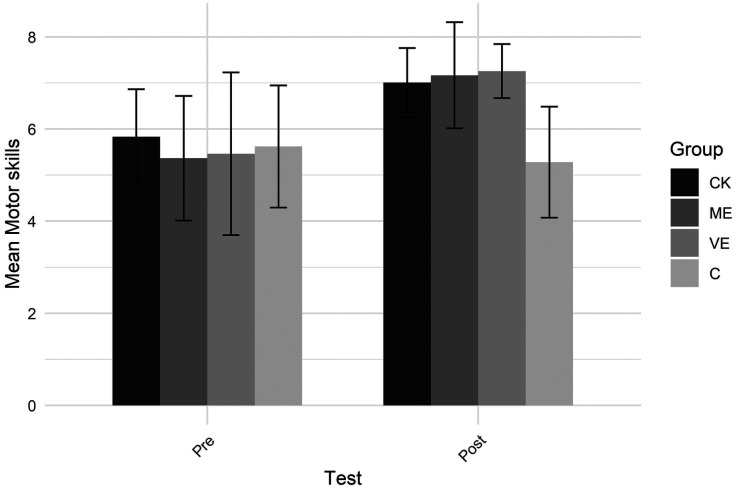
Motor skills.

Figure 8 shows self-evaluated cricket motor skills. A significant group by test interaction showed that perceived motor skills increased from pre to post-test only in the intervention groups (i.e., CK, ME and VE), not in the C group. At pre-test, the C group's motor skills were higher than the ME group's, but at post-test, they were lower than the VE group's and nearly lower than the ME group's. A main effect of group showed that the motor skills of the C group were lower than the CK and VE groups. Furthermore, a main effect of test showed that overall the motor skills increased from pre to post test.

#### 3.3.2 Content knowledge

**Figure 9:**
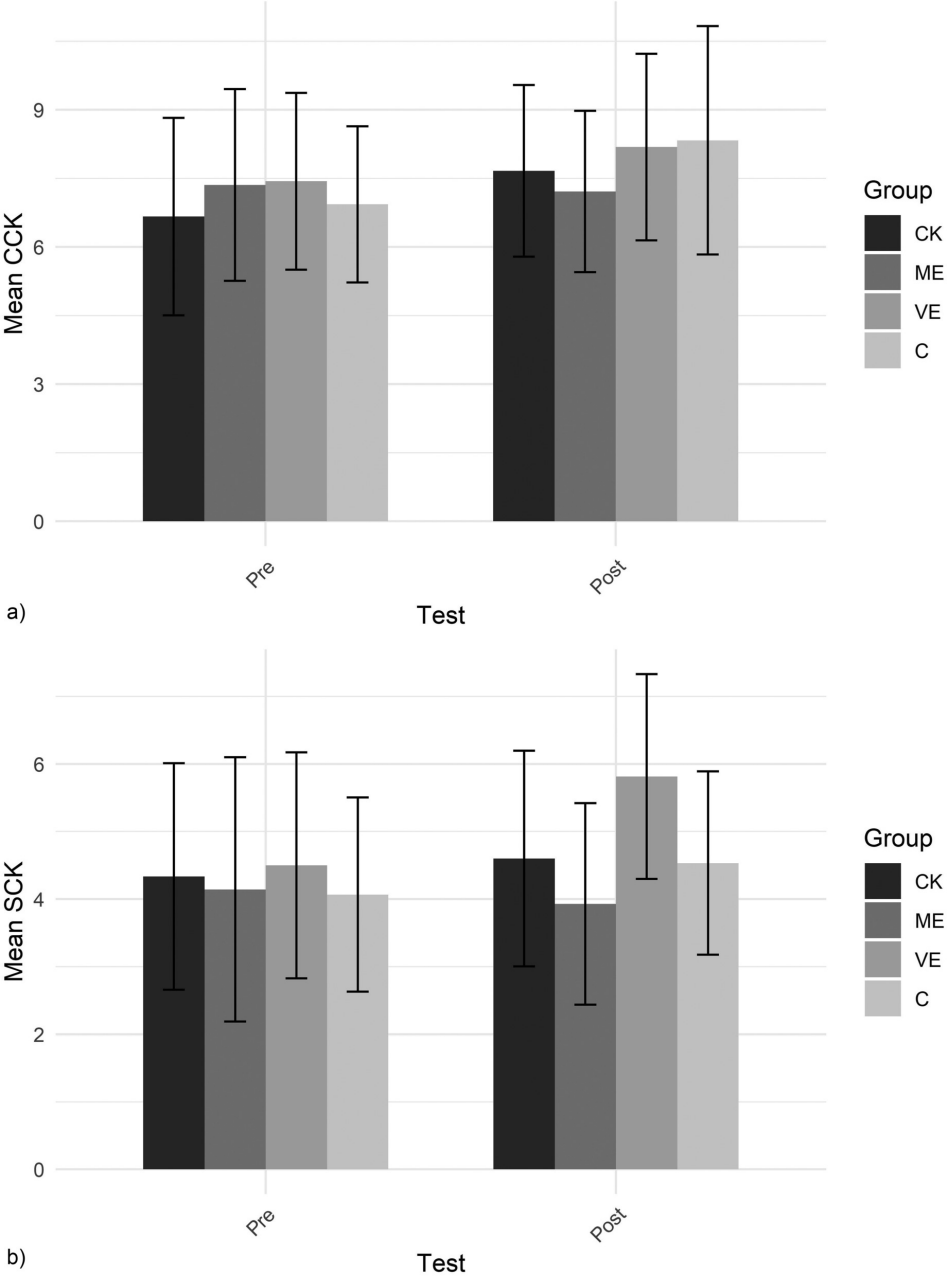
**(a)** CCK and **(b)** SCK.

The CCK and SCK scores are shown in Figure 9. *CCK* scores showed a marginally significant increase from pre- to post-test. *SCK* scores significantly increased from pre- to post-test.

## 4 Discussion

This study examined how three targeted educational programs, focused on content knowledge, motor experience, and visual experience, affected the perceptual skills of pre-service PE teachers. As hypothesized, pre-service teachers perceived a greater quantity, diversity and unpredictability of intervention affordances after the educational programs. Additionally, they demonstrated shorter times to intervention, a decreased quantity and diversity of guiding affordances, and a reduction in expressed uncertainty. These outcomes indicate that the pre-service teachers developed a more differentiated field of affordances.

A field of affordances is structured along three dimensions: scope (“width”), temporal horizon (“depth”), and relevance (“height”) (7, 20). The increased diversity of the perceived intervention affordances in the current study reflects a broader scope, or width, of the field of affordances. Furthermore, across all groups, education changed teaching behavior. Following education, pre-service teachers provided less verbal guidance but paused activities more often and earlier to act on perceived intervention affordances. These interventions became more varied and less predictable, reflecting a more adaptive teaching approach. This shift suggests that pre-service teachers not only improved their affordance perception but also gained a more strategic and certain teaching style, characterized by selective, impactful interventions.

Meanwhile, the faster times to intervention, the increased unpredictability of intervention affordances, and possibly also the reduced amount of expressed uncertainty, could collectively but cautiously be interpreted as signs of a more differentiated height of the field. As Aspbury-Miyanishi (67) argues a skilled teacher does not perceive a range of equally valid possibilities and then make a conscious, deliberate decision about which is best. Instead, they intuitively act on the most salient affordance, one that naturally compels them to act as it appears as the right course of action at that moment and in alignment with their intention(s). In contrast, an unskilled, novice teacher perceives a less differentiated height of the field. They perceive a, perhaps confusing, array of possibilities, none of which stands out as clearly appropriate. They may recognize information requiring a response but fail to perceive any actionable affordance associated with it. As a result, the novice teacher may pause, consciously think and deliberate over what to do by relying on principles, rules, or heuristics (57), possibly leading to slower times to intervention. Compared to novices, skilled teachers thus perceive a more differentiated height of the field of affordances, which implies closer contact with the environment and its possibilities (7). Following the educational program, the pre-service PE teachers appear better equipped to make adaptive, context-sensitive decisions, likely leading to more engaging and effective learning (56). The positive effects of all three education types on affordance perception underscore the importance of fostering this competency within PETE programs.

The findings also indicate increased teaching confidence after the educational program, shown by fewer statements of uncertainty. In PE settings, where quick decisions are vital for engagement and safety, greater perceptual confidence helps teachers to respond more promptly and convincingly. Confidence in teaching is vital for in-the-moment pedagogical decisions, such as deciding when to intervene, how to modify activities, or how to scaffold student learning for optimal engagement and skill progression. Importantly, students are often influenced—consciously or unconsciously—by the perceived confidence of their teacher, which can, in turn, shape their own behaviors and engagement (68, 69).

### 4.1 Group differences

While interpreting our results, caution is needed because the control group, which received no intervention, often performed similarly to the intervention groups (CK, ME, and VE). This suggests some changes might reflect habituation to the test, rather than direct effects of the educational programs. Nevertheless, a closer examination of the group-specific changes provides a more nuanced perspective. Only the ME group showed a significant increase in perceived teaching affordances from pre- to post-test, whereas the CK and VE groups did not exhibit a significant shift. Importantly, the C group displayed a trend towards a decrease in perceived affordances. Across groups, participants generally decreased their perception of guiding affordances while increasing their perception of intervention affordances from pre to posttest. However, the number of intervention affordances was significantly lower for the C group than for the ME and VE groups. There were also notable differences in the didactical categories of the teaching affordances. The VE group changed their didactical focus by increasing the percentage of perceived affordances categorized as facilitating learning and marginally reducing those for managing lessons from pre- to post-test. This change, from managing to facilitating, was also found more generally across groups, aligning with the overall shift from guiding to intervention affordances. In contrast, the C group tended to increase the percentage of affordances related to managing lessons. This collective pattern of divergent responses indicates that the C group's engagement with the teaching scenarios differed from that of the intervention groups. Large individual differences, short training time, and limitations of video-based assessments may have hidden some program effects (see limitations).

While all three targeted educational programs enhanced affordance perception, the specific program effects were nuanced. The program focused ME led to an increase in perceived teaching affordances from pre- to posttest. The VE program significantly altered the didactical category of the perceived teaching affordances: it specifically fostered an increase in those related to facilitating learning while reducing those concerning managing lessons. This observed shift matches with findings on professional vision from Reuker (35), who showed that PE teachers with high levels of pedagogical expertise noticed more methodological and didactical issues than novices, while both groups paid similar attention to management cues. Similarly, research on mathematics teachers has shown that with increasing experience, teachers' attention shifts from classroom organization and teacher instruction toward a deeper focus on understanding the content being taught (13, 15).

Although group differences were small, they may still matter. Small gains in affordance perception during a six-week course could grow into larger differences with extended training (70). In practice, perceiving a more differentiated field of teaching affordances gives teachers greater flexibility to adapt interventions to the ever-changing demands of teaching (7). Furthermore, expertise differences, at least in sports domains, tend to be more pronounced in more representative tasks (71), suggesting that group differences may be more evident in live teaching scenarios than in video-based assessments.

### 4.2 Educational program-specific insights

The three educational programs were designed to develop pre-service teachers' teaching skills through distinct approaches. The CK program focused on deepening pre-service teachers' understanding of cricket, covering common and specialized content knowledge, to help teachers better structure and scaffold student learning. The ME program emphasized practicing cricket skills to develop an embodied understanding of the movements, supporting the link between perception and action (39, 42). The VE program involved watching and analyzing cricket videos to expand perceptual experience and improve their ability to recognize more teaching affordances. Despite each program's specific goals, all groups improved in content knowledge, with no advantage for the CK group. Likewise, all intervention groups showed gains in perceived motor skills, but the ME group did not outperform the others. Similarly, improvements in gaze behavior occurred across all groups, with no distinct benefit for the VE group. This suggests that the improvements in content knowledge, perceived motor skills and gaze behavior likely resulted from general course activities (peer teaching) or test habituation, rather than the specific programs. This underscores the importance of grounding teacher education in authentic, context-rich experiences that reflect the dynamic interplay between teachers, learners, and the environment (56, 57), and aligns with an ecological perspective on perception (7, 72).

### 4.3 Gaze behavior

The gaze behavior measurements revealed that, after education, all groups showed fewer, longer fixations, indicating more focused visual search. This matches eye-tracking studies into teachers’ professional vision, that found that more experienced teachers direct their gaze more selectively and more purposefully than novices (73, 74), and aligns with prior research showing that, across domains, experts exhibit fewer, longer fixations than novices (75, 76). Similar gaze behavior patterns have been observed in sports coaches (77, 78). This change in gaze behavior may reflect a more deliberate and efficient visual search strategy.

No group differences were found in gaze behavior in the current study. Consequently, changes in gaze behavior cannot be attributed to the educational programs but are more likely a result of familiarity or experience with the testing procedure. However, education might have refined gaze strategies in ways our analyses missed. For example, other factors, such as fixation locations, preferred sequences and the timing of fixations on specific locations could differ across groups. Participants may have fixated for sufficient durations but on inappropriate locations, at suboptimal times, or in ineffective orders. Another limitation is that watching video limits perception compared to active, real-world interaction (79, 80).

### 4.4 Limitations and future directions

Several limitations should be addressed in future research. First, the short duration of the educational programs (five hours of targeted training) may have limited their overall impact, and appears minimal compared to the four-year full-time bachelor program in which pre-service teachers develop their teaching skills. We recommend that future research explore longer, more intensive interventions, and assess whether improvements transfer to teaching similar sports (e.g., softball). Second, video-based assessments may not fully capture real-world affordance perception. To better capture the dynamic interaction between teacher and environment, future studies should include live teaching assessments (57, 72). This would help move beyond video-based tasks and enhance the ecological validity of the findings. Furthermore, incorporating advanced methods, such as eye-tracking during live teaching, could provide deeper insights into gaze behavior and affordance perception. This may help refine training techniques and further enhance the development of perceptual skills of pre-service teachers.

Third, large individual differences were observed in the data, reflected by large standard deviations. While we controlled for prior cricket experience, variability in affordance perception and responses to the interventions may still be attributable to unmeasured individual factors, such as prior informal experience with related sports, cognitive styles, or learning preferences. To more rigorously assess the educational programs' efficacy, a large-scale randomized control trial is warranted.

Finally, longitudinal studies should examine whether improvements in affordance perception persist or grow as pre-service teachers continue their training or transition into in-service teaching roles. Additionally, future studies could explore the effects of different education sequences (e.g., CK before ME) or combinations of educational programs (e.g., CK + ME) to identify optimal approaches for PETE programs.

### 4.5 Practical applications

The findings have important implications for pre-service teacher education. Improved perception of teaching affordances in pre-service PE teachers supports more adaptive and responsive instructional strategies. This highlights the value of well-designed PETE programs. Many pre-service teachers initially focus on lesson management rather than on facilitating learning (35). Therefore, programs should actively promote this shift in focus, and the current study suggests that expanding visual experience is especially effective in supporting this transition. Such visual experience can be developed through video observation sessions, in which pre-service teachers analyze and discuss dynamic classroom scenarios. Similarly, “video clubs” have been shown to be beneficial for developing noticing or professional vision in mathematics teachers (13, 15, 16). Aligning with an ecological perspective on perception, which holds that skillful teaching involves perceiving and acting upon the opportunities for action (affordances) within the teaching-learning environment (7, 72), Aspbury-Miyanishi emphasizes that video can support teachers' perceptual learning when three key considerations are addressed (57). First, the videos should closely approximate the real teaching environment (e.g., featuring teacher's own practice from a first-person perspective). Second, verbal cues should be kept to a minimum, guiding attention to relevant information in the video without prescribing specific actions (e.g., “watch how student X responds” rather than instructing what to do about it). Third, because what teachers perceive and how they respond in actual teaching practice is most important, video sessions should be coupled with opportunities to respond in actual teaching practice. This allows teachers to attend to what was highlighted in the videos and reveal affordances in authentic situations. Thus, combining video-based learning with immersive, context-rich experiences supports a nuanced understanding of the real-time, dynamic interaction between teachers, learners, and the environment (56, 57).

## 5 Conclusion

This study examined how three targeted educational programs—CK, ME, and VE—affected perceptual and teaching skills of pre-service PE teachers. All groups improved intervention times, reduced uncertainty, and developed a more differentiated perception of affordances, essential for effective teaching in dynamic PE environments. Although group differences were subtle, the VE group showed a higher increase in affordances related to facilitating learning. Teaching behaviors improved across all groups, with participants demonstrating more selective and impactful interventions following education. These findings show that structured education can enhance adaptive and confident teaching. However, the short intervention duration and reliance on video-based assessments limited ecological validity. Future research should explore longer interventions and real-world teaching contexts. Integrating CK, ME, and VE into PETE programs can equip pre-service teachers with the skills needed for adaptive, effective instruction, ultimately enhancing PE learning experiences.

## Data Availability

The raw data supporting the conclusions of this article will be made available by the authors, without undue reservation.
