# Supplementary material for: Enhancing affordance perception in pre-service physical education teachers: effects of content knowledge, motor experience and visual experience programs
Source: Front Sports Act Living. 2025 Jul 2;7:1583448. doi: 10.3389/fspor.2025.1583448 (PMC12263654; doi:10.3389/fspor.2025.1583448)
Supplement: Supplementary file 1 [file Datasheet1.pdf]

Supplementary Table 1. CONSORT checklist

Section/topic	No	CONSORT 2025 checklist item description	Reported on page no.
**Title and abstract**			
Title and structured abstract	1a	Identification as a randomised trial	Not applicable
	1b	Structured summary of the trial design, methods, results, and conclusions	p. 1
**Open science**			
Trial registration	2	Name of trial registry, identifying number (with URL) and date of registration	Not applicable
Protocol and statistical analysis plan	3	Where the trial protocol and statistical analysis plan can be accessed	Not applicable
Data sharing	4	Where and how the individual de-identified participant data (including data dictionary), statistical code and any other materials can be accessed	p.18
Funding and conflicts of interest	5a	Sources of funding and other support (eg, supply of drugs), and role of funders in the design, conduct, analysis and reporting of the trial	p. 18
	5b	Financial and other conflicts of interest of the manuscript authors	p. 18
**Introduction**			
Background and rationale	6	Scientific background and rationale	p. 2-4
Objectives	7	Specific objectives related to benefits and harms	p. 2-4
**Methods**			
Patient and public involvement	8	Details of patient or public involvement in the design, conduct and reporting of the trial	Not applicable
Trial design	9	Description of trial design including type of trial (eg, parallel group, crossover), allocation ratio, and framework (eg, superiority, equivalence, non-inferiority, exploratory)	p. 4
Changes to trial protocol	10	Important changes to the trial after it commenced including any outcomes or analyses that were not prespecified, with reason	p. 5 & 10
Trial setting	11	Settings (eg, community, hospital) and locations (eg, countries, sites) where the trial was conducted	p. 4-5
Eligibility criteria	12a	Eligibility criteria for participants	p. 4
	12b	If applicable, eligibility criteria for sites and for individuals delivering the interventions (eg, surgeons, physiotherapists)	p. 5
Intervention and comparator	13	Intervention and comparator with sufficient details to allow replication. If relevant, where additional materials describing the intervention and comparator (eg, intervention manual) can be accessed	p. 5 & Table 2
Outcomes	14	Prespecified primary and secondary outcomes, including the specific measurement variable (eg, systolic blood pressure), analysis metric (eg, change from baseline, final value, time to event), method of aggregation (eg, median, proportion), and time point for each outcome	p. 5-8
Harms	15	How harms were defined and assessed (eg, systematically, non-systematically)	Not applicable
Sample size	16a	How sample size was determined, including all assumptions supporting the sample size calculation	Not applicable
	16b	Explanation of any interim analyses and stopping guidelines	Not applicable
Randomisation:			
Sequence generation	17a	Who generated the random allocation sequence and the method used	Not applicable
	17b	Type of randomisation and details of any restriction (eg, stratification, blocking and block size)	Not applicable

			Reported on page no.
Allocation concealment mechanism	18	Mechanism used to implement the random allocation sequence (eg, central computer/telephone; sequentially numbered, opaque, sealed containers), describing any steps to conceal the sequence until interventions were assigned	Not applicable
Implementation	19	Whether the personnel who enrolled and those who assigned participants to the interventions had access to the random allocation sequence	Not applicable
Blinding	20a	Who was blinded after assignment to interventions (eg, participants, care providers, outcome assessors, data analysts)	Not applicable
	20b	If blinded, how blinding was achieved and description of the similarity of interventions	Not applicable
Statistical methods	21a	Statistical methods used to compare groups for primary and secondary outcomes, including harms	p. 8
	21b	Definition of who is included in each analysis (eg, all randomised participants), and in which group	p. 4 & Table 1
	21c	How missing data were handled in the analysis	Not applicable
	21d	Methods for any additional analyses (eg, subgroup and sensitivity analyses), distinguishing prespecified from post hoc	p. 10
**Results**			
Participant flow, including flow diagram	22a	For each group, the numbers of participants who were randomly assigned, received intended intervention, and were analysed for the primary outcome	Table 1
	22b	For each group, losses and exclusions after randomisation, together with reasons	Not applicable
Recruitment	23a	Dates defining the periods of recruitment and follow-up for outcomes of benefits and harms	
	23b	If relevant, why the trial ended or was stopped	Not applicable
Intervention and comparator delivery	24a	Intervention and comparator as they were actually administered (eg, where appropriate, who delivered the intervention/comparator, how participants adhered, whether they were delivered as intended (fidelity))	p. 5 & Table 2
	24b	Concomitant care received during the trial for each group	Not applicable
Baseline data	25	A table showing baseline demographic and clinical characteristics for each group	Table 1
Numbers analysed, outcomes and estimation	26	For each primary and secondary outcome, by group:<br>● the number of participants included in the analysis<br>● the number of participants with available data at the outcome time point<br>● result for each group, and the estimated effect size and its precision (such as 95% confidence interval)<br>● for binary outcomes, presentation of both absolute and relative effect size	p. 8-10, Table 4, Figures 1-9
Harms	27	All harms or unintended events in each group	Not applicable
Ancillary analyses	28	Any other analyses performed, including subgroup and sensitivity analyses, distinguishing pre-specified from post hoc	p. 10
**Discussion**			
Interpretation	29	Interpretation consistent with results, balancing benefits and harms, and considering other relevant evidence	p. 10-18
Limitations	30	Trial limitations, addressing sources of potential bias, imprecision, generalisability, and, if relevant, multiplicity of analyses	p. 10-18

Hopewell S, Chan AW, Collins GS, Hróbjartsson A, Moher D, Schulz KF, et al. CONSORT 2025 Statement: updated guideline for reporting randomised trials. BMJ. 2025; 388:e081123.
https://dx.doi.org/10.1136/bmj-2024-081123

Supplementary Table 2. Post hoc pairwise comparisons.

DV	Term		Pairwise comparison	estimate	SE	df	lower.CL	upper.CL	t	p
Time to intervention										
	Test		Post - Pre	-21.6	3.6	56	-28.8	-14.4	-6.005	<.001
Number of perceived teaching affordances										
	Group		C - CK	29.4	15.4	56	-11.4	70.2	1.907	0.237
			C - ME	44.5	15.7	56	2.9	86.0	2.833	0.032
			C - VE	33.0	15.2	56	-7.2	73.2	2.174	0.143
			CK - ME	15.1	15.7	56	-26.5	56.6	0.959	0.773
			CK - VE	3.6	15.2	56	-36.6	43.8	0.236	0.995
			ME - VE	-11.5	15.4	56	-52.4	29.4	-0.742	0.880
	Test		post - pre	-34.0	4.4	56	-42.9	-25.1	-7.675	<.001
	Type		Guiding - Intervention	47.3	11.0	56	25.4	69.3	4.321	0.000
	Group x Test	Post:	C - CK	-8.8	16.9	76	-53.1	35.6	-0.519	0.954
			C - ME	-20.9	17.2	76	-66.0	24.2	-1.216	0.619
			C - VE	-2.8	16.6	76	-46.5	40.9	-0.169	0.998
			CK - ME	-12.1	17.2	76	-57.3	33.0	-0.706	0.894
			CK - VE	6.0	16.6	76	-37.7	49.6	0.359	0.984
			ME - VE	18.1	16.9	76	-26.4	62.6	1.070	0.709
		Pre:	C - CK	15.2	16.9	76	-29.2	59.6	0.900	0.805
			C - ME	20.2	17.2	76	-25.0	65.3	1.174	0.645
			C - VE	2.2	16.6	76	-41.5	45.8	0.130	0.999
			CK - ME	5.0	17.2	76	-40.2	50.1	0.289	0.992
			CK - VE	-13.0	16.6	76	-56.7	30.6	-0.784	0.861
			ME - VE	-18.0	16.9	76	-62.5	26.4	-1.064	0.712
		C:	post - pre	-15.9	9.4	56	-34.8	3.0	-1.690	0.097
		CK:	post - pre	8.0	9.4	56	-10.9	26.9	0.852	0.398
		ME:	post - pre	25.1	9.8	56	5.6	44.7	2.577	0.013
		VE:	post - pre	-11.0	9.1	56	-29.3	7.3	-1.202	0.235
	Group x Type	Guiding:	C - CK	19.5	21.8	112	-37.5	76.4	0.892	0.809
			C - ME	25.3	22.2	112	-32.6	83.2	1.139	0.666
			C - VE	21.3	21.5	112	-34.7	77.3	0.991	0.755
			CK - ME	5.8	22.2	112	-52.1	63.8	0.263	0.994
			CK - VE	1.8	21.5	112	-54.2	57.9	0.085	1.000
			ME - VE	-4.0	21.9	112	-61.1	53.1	-0.183	0.998
		Intervention:	C - CK	-42.6	21.8	112	-99.5	14.3	-1.952	0.213
			C - ME	-79.2	22.2	112	-137.1	-21.2	-3.563	0.003
			C - VE	-70.7	21.5	112	-126.8	-14.7	-3.292	0.007
			CK - ME	-36.6	22.2	112	-94.5	21.4	-1.646	0.358
			CK - VE	-28.1	21.5	112	-84.2	27.9	-1.310	0.559
			ME - VE	8.4	21.9	112	-48.6	65.5	0.385	0.981
		C:	Guiding - Intervention	31.0	22.4	56	-13.9	75.9	1.383	0.172
		CK:	Guiding - Intervention	-31.1	22.4	56	-76.0	13.8	-1.386	0.171
		ME:	Guiding - Intervention	-73.5	23.2	56	-120.0	-27.0	-3.166	0.003
		VE:	Guiding - Intervention	-61.0	21.7	56	-104.5	-17.5	-2.812	0.007
	Test x Type	Guiding:	post - pre	-19.3	6.7	108	-32.6	-6.0	-2.882	0.005
		Intervention:	post - pre	61.8	6.7	108	48.5	75.1	9.227	<.001
		Post:	Guiding - Intervention	-76.0	12.3	79	-100.5	-51.5	-6.183	<.001
		Pre:	Guiding - Intervention	5.1	12.3	79	-19.3	29.6	0.417	0.678
Number of unique teaching affordances										
	Test x Type	Guiding:	post - pre	-26.1	7.6	108	-41.1	-11.0	-3.423	0.001
		Intervention:	post - pre	25.2	7.6	108	10.1	40.3	3.312	0.001
		Post:	Guiding - Intervention	-19.6	12.9	83	-45.4	6.1	-1.516	0.133
		Pre:	Guiding - Intervention	31.6	12.9	83	5.9	57.4	2.442	0.017
Shannon entropy										
	Test x Type	Guiding:	post - pre	-20.7	8.4	86	-37.5	-4.0	-2.457	0.016
		Intervention:	post - pre	17.2	8.4	86	0.4	34.0	2.036	0.045
		Post:	Guiding - Intervention	-10.3	11.1	83	-32.3	11.8	-0.928	0.356
		Pre:	Guiding - Intervention	27.6	11.1	83	5.6	49.7	2.493	0.015
Teaching affordance didactical categories										
	Category		Facilitating learning - Guiding exp	126.6	17.8	112	84.3	168.9	7.109	<.001
			Facilitating learning - Managing les	95.8	17.8	112	53.5	138.1	5.383	<.001
			Guiding experiences - Managing le	-30.7	17.8	112	-73.0	11.5	-1.727	0.200
	Group x Category	Facilitating learining:	C - CK	-74.1	33.1	116	-160.4	12.2	-2.239	0.119
			C - ME	-32.9	33.7	116	-120.7	54.9	-0.976	0.763
			C - VE	-92.4	32.6	116	-177.3	-7.4	-2.835	0.027
			CK - ME	41.2	33.7	116	-46.6	129.1	1.224	0.613
			CK - VE	-18.2	32.6	116	-103.2	66.7	-0.560	0.944
			ME - VE	-59.5	33.2	116	-146.0	27.0	-1.793	0.282
		Guiding experiences:	C - CK	55.3	33.1	116	-31.0	141.6	1.671	0.344
			C - ME	81.5	33.7	116	-6.3	169.3	2.419	0.079
			C - VE	75.5	32.6	116	-9.5	160.4	2.316	0.100
			CK - ME	26.2	33.7	116	-61.7	114.0	0.777	0.865
			CK - VE	20.1	32.6	116	-64.8	105.1	0.618	0.926
			ME - VE	-6.0	33.2	116	-92.5	80.5	-0.182	0.998
		Managing lessons:	C - CK	44.8	33.1	116	-41.5	131.1	1.353	0.531
			C - ME	-40.1	33.7	116	-127.9	47.8	-1.189	0.635
			C - VE	17.5	32.6	116	-67.4	102.5	0.538	0.950

		CK - ME	-84.9	33.7	116	-172.7	3.0	-2.518	0.062
		CK - VE	-27.3	32.6	116	-112.2	57.7	-0.836	0.837
		ME - VE	57.6	33.2	116	-28.9	144.1	1.736	0.310
	C:	Facilitating learning - Guiding exp	-105.5	40.2	112	-200.8	-10.1	-2.627	0.026
		Facilitating learning - Managing le	-62.2	40.2	112	-157.6	33.2	-1.549	0.272
		Guiding experiences - Managing le	43.3	40.2	112	-52.1	138.6	1.078	0.530
	CK:	Facilitating learning - Guiding exp	24.0	40.2	112	-71.4	119.4	0.598	0.822
		Facilitating learning - Managing le	56.7	40.2	112	-38.6	152.1	1.413	0.338
		Guiding experiences - Managing le	32.7	40.2	112	-62.6	128.1	0.815	0.694
	ME:	Facilitating learning - Guiding exp	8.9	41.6	112	-89.8	107.7	0.215	0.975
		Facilitating learning - Managing le	-69.4	41.6	112	-168.1	29.4	-1.669	0.222
		Guiding experiences - Managing le	-78.3	41.6	112	-177.0	20.4	-1.884	0.148
	VE:	Facilitating learning - Guiding exp	62.4	38.9	112	-30.0	154.7	1.604	0.248
		Facilitating learning - Managing le	47.7	38.9	112	-44.6	140.1	1.227	0.440
		Guiding experiences - Managing le	-14.7	38.9	112	-107.0	77.7	-0.377	0.925
Test x Category	Facilitating learining:	Post - pre	59.3	12.8	117	33.9	84.7	4.620	<.001
	Guiding experiences:	Post - pre	-18.5	12.8	117	-43.9	6.9	-1.441	0.152
	Managing lessons:	Post - pre	-44.1	12.8	117	-69.6	-18.7	-3.439	0.001
	Post:	Facilitating learning - Guiding exp	34.0	23.3	171	-21.0	89.0	1.460	0.313
		Facilitating learning - Managing le	45.6	23.3	171	-9.4	100.7	1.961	0.125
		Guiding experiences - Managing le	11.6	23.3	171	-43.4	66.7	0.501	0.871
	Pre:	Facilitating learning - Guiding exp	-43.8	23.3	171	-98.9	11.2	-1.884	0.147
		Facilitating learning - Managing le	-57.8	23.3	171	-112.9	-2.8	-2.485	0.037
		Guiding experiences - Managing le	-14.0	23.3	171	-69.0	41.0	-0.602	0.819
Group x Test x Category	Post: Facilitating learin	C - CK	17.2	38.7	177	-83.1	117.4	0.444	0.971
		C - ME	-27.1	39.3	177	-129.1	75.0	-0.688	0.902
		C - VE	-54.2	38.0	177	-152.9	44.5	-1.425	0.486
		CK - ME	-44.2	39.3	177	-146.2	57.8	-1.124	0.675
		CK - VE	-71.4	38.0	177	-170.0	27.3	-1.876	0.242
		ME - VE	-27.2	38.7	177	-127.6	73.3	-0.701	0.897
	Pre: Facilitating leariniı	C - CK	24.1	38.7	177	-76.2	124.3	0.623	0.925
		C - ME	36.9	39.3	177	-65.1	138.9	0.938	0.785
		C - VE	52.4	38.0	177	-46.3	151.0	1.376	0.516
		CK - ME	12.8	39.3	177	-89.2	114.8	0.326	0.988
		CK - VE	28.3	38.0	177	-70.4	126.9	0.743	0.879
		ME - VE	15.5	38.7	177	-85.0	115.9	0.399	0.978
	Post: Guiding experien	C - CK	-18.8	38.7	177	-119.0	81.4	-0.486	0.962
		C - ME	-10.3	39.3	177	-112.3	91.8	-0.261	0.994
		C - VE	0.4	38.0	177	-98.3	99.1	0.011	1.000
		CK - ME	8.5	39.3	177	-93.5	110.6	0.217	0.996
		CK - VE	19.2	38.0	177	-79.5	117.9	0.505	0.958
		ME - VE	10.7	38.7	177	-89.8	111.1	0.275	0.993
	Pre: Guiding experienc	C - CK	11.3	38.7	177	-88.9	111.5	0.292	0.991
		C - ME	0.5	39.3	177	-101.5	102.5	0.013	1.000
		C - VE	-11.0	38.0	177	-109.7	87.6	-0.290	0.992
		CK - ME	-10.8	39.3	177	-112.8	91.2	-0.274	0.993
		CK - VE	-22.3	38.0	177	-121.0	76.3	-0.587	0.936
		ME - VE	-11.6	38.7	177	-112.0	88.9	-0.298	0.991
	Post: managing lessons	C - CK	-0.4	38.7	177	-100.6	99.8	-0.010	1.000
		C - ME	34.7	39.3	177	-67.3	136.7	0.882	0.814
		C - VE	36.2	38.0	177	-62.5	134.9	0.952	0.777
		CK - ME	35.1	39.3	177	-66.9	137.1	0.892	0.809
		CK - VE	36.6	38.0	177	-62.1	135.3	0.962	0.771
		ME - VE	1.5	38.7	177	-98.9	102.0	0.039	1.000
	Pre: Managing lessons:	C - CK	-22.2	38.7	177	-122.4	78.0	-0.574	0.940
		C - ME	-37.6	39.3	177	-139.6	64.4	-0.956	0.774
		C - VE	-50.6	38.0	177	-149.3	48.1	-1.330	0.545
		CK - ME	-15.4	39.3	177	-117.4	86.6	-0.392	0.980
		CK - VE	-28.4	38.0	177	-127.1	70.3	-0.746	0.878
		ME - VE	-13.0	38.7	177	-113.4	87.5	-0.335	0.987
	C: Facilitating leariniη	post - pre	-34.9	25.8	118	-86.0	16.3	-1.349	0.180
	CK: Facilitating learini	post - pre	-28.0	25.8	118	-79.1	23.2	-1.082	0.281
	ME: Facilitating learini	post - pre	29.1	26.7	118	-23.9	82.0	1.087	0.279
	VE: Facilitating learini	post - pre	71.7	25.0	118	22.1	121.2	2.865	0.005
	C: Guiding experiences	post - pre	-7.5	25.8	118	-58.7	43.7	-0.290	0.772
	CK: Guiding experienc	post - pre	22.6	25.8	118	-28.6	73.8	0.875	0.384
	ME: Guiding experienc	post - pre	3.3	26.7	118	-49.7	56.3	0.123	0.902
	VE: Guiding experienc	post - pre	-18.9	25.0	118	-68.5	30.6	-0.757	0.451
	C: Managing lessons:	post - pre	43.9	25.8	118	-7.3	95.0	1.698	0.092
	CK: Managing lessons:	post - pre	22.1	25.8	118	-29.1	73.2	0.854	0.395
	ME: Managing lessons	post - pre	-28.4	26.7	118	-81.4	24.5	-1.063	0.290
	VE: Managing lessons:	post - pre	-42.9	25.0	118	-92.5	6.6	-1.716	0.089
	C: Post:	Facilitating learning - Guiding exp	-9.9	46.9	171	-120.8	101.0	-0.210	0.976
		Facilitating learning - Managing le	-35.7	46.9	171	-146.6	75.2	-0.760	0.728
		Guiding experiences - Managing le	-25.8	46.9	171	-136.7	85.1	-0.550	0.847
	CK: Post:	Facilitating learning - Guiding exp	-45.8	46.9	171	-156.7	65.1	-0.977	0.592
		Facilitating learning - Managing le	-53.2	46.9	171	-164.1	57.7	-1.135	0.494
		Guiding experiences - Managing le	-7.4	46.9	171	-118.3	103.5	-0.158	0.986
	ME: Post:	Facilitating learning - Guiding exp	6.9	48.6	171	-107.9	121.7	0.143	0.989
		Facilitating learning - Managing le	26.1	48.6	171	-88.7	140.9	0.537	0.853
		Guiding experiences - Managing le	19.1	48.6	171	-95.6	133.9	0.394	0.918
	VE: Post:	Facilitating learning - Guiding exp	44.8	45.4	171	-62.6	152.1	0.985	0.587
		Facilitating learning - Managing le	54.8	45.4	171	-52.6	162.1	1.206	0.452

			Guiding experiences - Managing le	10.0	45.4	171	-97.4	117.4	0.220	0.974
		C: Pre:	Facilitating learning - Guiding exp	17.5	46.9	171	-93.4	128.4	0.373	0.926
			Facilitating learning - Managing le	43.1	46.9	171	-67.8	154.0	0.918	0.630
			Guiding experiences - Managing le	25.6	46.9	171	-85.3	136.5	0.545	0.849
		CK: Pre:	Facilitating learning - Guiding exp	4.7	46.9	171	-106.2	115.6	0.101	0.994
			Facilitating learning - Managing le	-3.2	46.9	171	-114.1	107.7	-0.068	0.997
			Guiding experiences - Managing le	-7.9	46.9	171	-118.8	103.0	-0.169	0.984
		ME: Pre:	Facilitating learning - Guiding exp	-18.9	48.6	171	-133.6	95.9	-0.388	0.920
			Facilitating learning - Managing le	-31.4	48.6	171	-146.2	83.4	-0.647	0.794
			Guiding experiences - Managing le	-12.6	48.6	171	-127.4	102.2	-0.259	0.964
		VE: Pre:	Facilitating learning - Guiding exp	-45.9	45.4	171	-153.3	61.5	-1.010	0.572
			Facilitating learning - Managing le	-59.9	45.4	171	-167.3	47.5	-1.318	0.387
			Guiding experiences - Managing le	-14.0	45.4	171	-121.4	93.4	-0.308	0.949
Uncertainty										
	Test		post - pre	-25.7	4.4	56	-34.6	-16.9	-5.837	<.001
Search rate										
	Test		Post - Pre	-13.0	4.7	56	-22.4	-3.6	-2.767	0.008
Fixation duration										
	Test		Post - Pre	15.9	3.8	56	8.3	23.5	4.185	0.000
Motor skills										
	Group		C - CK	-30.0	10.0	56	-56.4	-3.6	-3.010	0.020
			C - ME	-23.1	10.1	56	-49.9	3.8	-2.274	0.116
			C - VE	-30.6	9.8	56	-56.5	-4.6	-3.116	0.015
			CK - ME	6.9	10.1	56	-19.9	33.8	0.683	0.903
			CK - VE	-0.6	9.8	56	-26.5	25.4	-0.058	1.000
			ME - VE	-7.5	10.0	56	-33.9	19.0	-0.750	0.876
	Test		Post - Pre	31.6	4.5	56	22.6	40.5	7.073	<.001
	Group x Test	Post:	C - CK	-21.5	12.0	100	-53.0	9.9	-1.788	0.285
			C - ME	-29.6	12.3	100	-61.6	2.4	-2.415	0.081
			C - VE	-32.7	11.9	100	-63.6	-1.7	-2.756	0.035
			CK - ME	-8.1	12.3	100	-40.1	24.0	-0.658	0.913
			CK - VE	-11.1	11.9	100	-42.1	19.8	-0.939	0.784
			ME - VE	-3.1	12.1	100	-34.6	28.5	-0.254	0.994
		Pre:	C - CK	26.5	12.0	100	-4.9	58.0	2.203	0.129
			C - ME	33.8	12.3	100	1.8	65.8	2.758	0.034
			C - VE	28.6	11.9	100	-2.4	59.5	2.409	0.082
			CK - ME	7.3	12.3	100	-24.8	39.3	0.593	0.934
			CK - VE	2.0	11.9	100	-29.0	33.0	0.170	0.998
			ME - VE	-5.3	12.1	100	-36.8	26.3	-0.435	0.972
		C:	Post - Pre	-51.5	9.7	56	-70.9	-32.0	-5.309	<.001
		CK:	Post - Pre	-3.4	9.7	56	-22.8	16.0	-0.351	0.727
		ME:	Post - Pre	11.9	10.0	56	-8.2	32.0	1.189	0.240
		VE:	Post - Pre	9.8	9.4	56	-9.1	28.6	1.039	0.303
SCK										
	Test		Post - Pre	11.3	5.5	56	0.2	22.4	2.047	0.045
